# The Aquaporin Gene Family of the Yellow Fever Mosquito, *Aedes aegypti*

**DOI:** 10.1371/journal.pone.0015578

**Published:** 2010-12-29

**Authors:** Lisa L. Drake, Dmitri Y. Boudko, Osvaldo Marinotti, Victoria K. Carpenter, Angus L. Dawe, Immo A. Hansen

**Affiliations:** 1 Department of Biology, New Mexico State University, Las Cruces, New Mexico, United States of America; 2 Institute of Applied Biosciences, New Mexico State University, Las Cruces, New Mexico, United States of America; 3 Molecular Biology Program, New Mexico State University, Las Cruces, New Mexico, United States of America; 4 Chicago Medical School, Rosalind Franklin University, North Chicago, Illinois, United States of America; 5 Department of Molecular Biology and Biochemistry, University of California Irvine, Irvine, California, United States of America; Universidade Federal do Rio de Janeiro, Brazil

## Abstract

**Background:**

The mosquito, *Aedes aegypti*, is the principal vector of the Dengue and yellow fever viruses. During feeding, an adult female can take up more than its own body weight in vertebrate blood. After a blood meal females excrete large amounts of urine through their excretion system, the Malpighian tubules (MT). Diuresis starts within seconds after the mosquito starts feeding. Aquaporins (AQPs) are a family of membrane transporters that regulate the flow of water, glycerol and other small molecules across cellular membranes in both prokaryotic and eukaryotic cells. Our aim was to identify aquaporins that function as water channels, mediating transcellular water transport in MTs of adult female *Ae. aegypti*.

**Methodology/Principal Findings:**

Using a bioinformatics approach we screened genome databases and identified six putative AQPs in the genome of *Ae. aegypti*. Phylogenetic analysis showed that five of the six *Ae. aegypti* AQPs have high similarity to classical water-transporting AQPs of vertebrates. Using microarray, reverse transcription and real time PCR analysis we found that all six AQPs are expressed in distinct patterns in mosquito tissues/body parts. AaAQP1, 4, and 5 are strongly expressed in the adult female MT. RNAi-mediated knockdown of the MT-expressed mosquito AQPs resulted in significantly reduced diuresis.

**Conclusions/Significance:**

Our results support the notion that AQP1, 4, and 5 function as water transporters in the MTs of adult female *Ae. aegypti* mosquitoes. Our results demonstrate the importance of these AQPs for mosquito diuresis after blood ingestion and highlight their potential as targets for the development of novel vector control strategies.

## Introduction

Anautogenous mosquito females need vertebrate blood for reproduction. The nutrients taken up with the blood are used to synthesize large amounts of yolk proteins that are deposited in the eggs during a process called vitellogenesis. Yolk delivers the energy and building blocks for embryogenesis. This “need for blood” in order to reproduce makes anautogenous mosquitoes effective disease vectors because they require at least one insect-host contact for every batch of eggs they develop.

During feeding, adult female *Aedes aegypti*, can take up more than their own body weight in blood [1]. This severely impairs their mobility and puts them at risk to be killed by their host or other predators. In addition, mosquitoes face the problem of high sodium content of vertebrate blood plasma and high potassium content in red blood cells. Therefore, it is essential for mosquitoes to possess an efficient system for excretion of excess water and ions while retaining the nutrients contained in the blood meal. During and after a blood meal female *Ae. aegypti* secrete large amounts of urine through their Malpighian tubules (MT). Within the first hour after taking a blood meal (post blood meal - PBM) mosquito females can discharge more than 40% of water and sodium enclosed in the blood plasma [1]. Diuresis is under hormonal control by neuropeptide hormones secreted by the central nervous system [2]. In the current model, diuretic hormones, released seconds after start of the blood meal, stimulate the MT cells to produce the second messenger molecule cAMP which activates transcellular diuresis by increasing transepithelial cation (Na^+^, K^+^) transport [2]. Another class of neuropeptide hormones, the kinins, increase intracellular calcium levels that regulate anion movement (Cl^−^) into the MT lumen [3], [4]. Urine produced by the MT is collected in the hind gut and subsequently forcefully ejected from the rectum in a process that involves rectal peristalsis and movement of the 7^th^ and 8^th^ abdominal segments. Females start expelling small urine droplets approximately 50–75 seconds after start of feeding. Urine droplets have a volume of about 10 to 12 nl and can fly up to 10 mm [1], [5].

Aquaporins (AQPs) are transport channels that make cell membranes permeable to water. They are found in all plant, animal, fungi, eubacteria, and archaea taxa studied [6], [7], [8]. In mammals, there are 13 AQPs and they form two subfamilies with different transport selectivity: orthodox aquaporins, which transport only water, and aquaglyceroporins, which transport glycerol, urea, small solutes, and water [9]. In the so-called hourglass model for AQP structure, the six transmembrane alpha helical domains (numbered 1 – 6) are connected by five loops termed A – E [10], [11], [12]. Both amino- and carboxy-terminus are located inside the cytoplasm. The transmembrane domains 2–3 and 5–6 are connected by loops B and E, both containing a highly conserved NPA (Asparagine-Proline-Alanine) motive and other conserved residues. These hydrophobic NPA loops form a ring as part of an hourglass-shaped pore within the center of the phospholipid bilayer membrane. This ring, with a diameter of 2.8 Å, is the primary filter that prevents protons from crossing through the AQP pore. Hg^2+^ ions interact with a cysteine residue close to the NPA motive in the E loop and an alanine residue in the B loop of most AQPs and efficiently obstruct water transport through the pore [13].

The activity of eukaryotic aquaporins is commonly regulated via three different mechanisms: translation, gating, or trafficking [13]. While regulation via translation is a relatively slow process, gating and trafficking can change water permeability of a membrane within seconds. Trafficking of aquaporins was first described in AQP2 in mammals where it is involved in concentrating urine in the kidneys [14]. AQP2-trafficking is controlled by a signaling cascade triggered by the neuropeptide arginine-vasopressin. The phosphorylation of conserved serine and theronine residues in the fourth loop region of AQP2 caused the redistribution of intracellular AQP storage vesicles to the plasma membrane. This resulted in a rapid increase of water permeability of the membrane. The mechanism of how AQP phosphorylation leads to the recognition of the flagged protein and subsequent vesicle movement and membrane fusion is still unknown. Gating refers to the ability of AQPs to control the flux of water by widening or constricting the channel. X-ray structures have revealed that the width of a fully opened channel can allow a single water molecule entry [13].

While vertebrate AQPs are well studied, few studies have been conducted on invertebrate AQPs (reviewed by Spring et al., 2009 [15]). DRIP (Genbank accession #: CG9023) is a partly characterized representative of *Drosophila* AQPs. It is expressed in embryonic and adult MTs of the fruit fly [16]. A mosquito AQP, a close homologue to *Drosophila* DRIP, has been cloned and characterized in the yellow fever mosquito *Ae. aegypti*. This AQP is localized in tracheolar cells associated with MTs of adult female *Ae. aegypti* mosquitoes [17]. Another mosquito aquaporin has recently been cloned and characterized from *Anopheles gambiae.* This aquaporin is a water transporter and expressed in multiple tissue and specifically in the stellate cells of MTs. It is important for water homeostasis in *An. gambiae* [18].

In order to evaluate the potential of this class of molecule as targets for vector control strategies, we have surveyed the genome of *Ae. aegypti* and identified six genes encoding putative AQPs. We show that four AQPs are expressed in the Malpighian tubules of adult females and that knockdown of three of them affects diuresis.

## Results

### The AQP genes of *Ae. aegypti*

BLAST searches conducted with vertebrate and *Drosophila* AQPs as query sequences allowed the identification of six loci encoding putative AQPs in the genome of *Ae. aegypti*, which were denominated *Ae. aegypti* aquaporins 1–6 (Table S1; AaAQP1–6). In addition we identified seven putative AQPs encoded in the genome of the malaria mosquito *Anopheles gambiae* [19], eight in the *Drosophila* genome [20], six in the genomic sequence of the head louse *Pediculus humanus* [21], and seven in the genome of the red flour beetle *Triboleum castaneum* [22], [23]. We also used five putative AQP of *Leishmania major*, six *Plasmodium*, and two fungal sequences of the yeast *Pichia pastoris* for our analysis.

**Figure 1:**
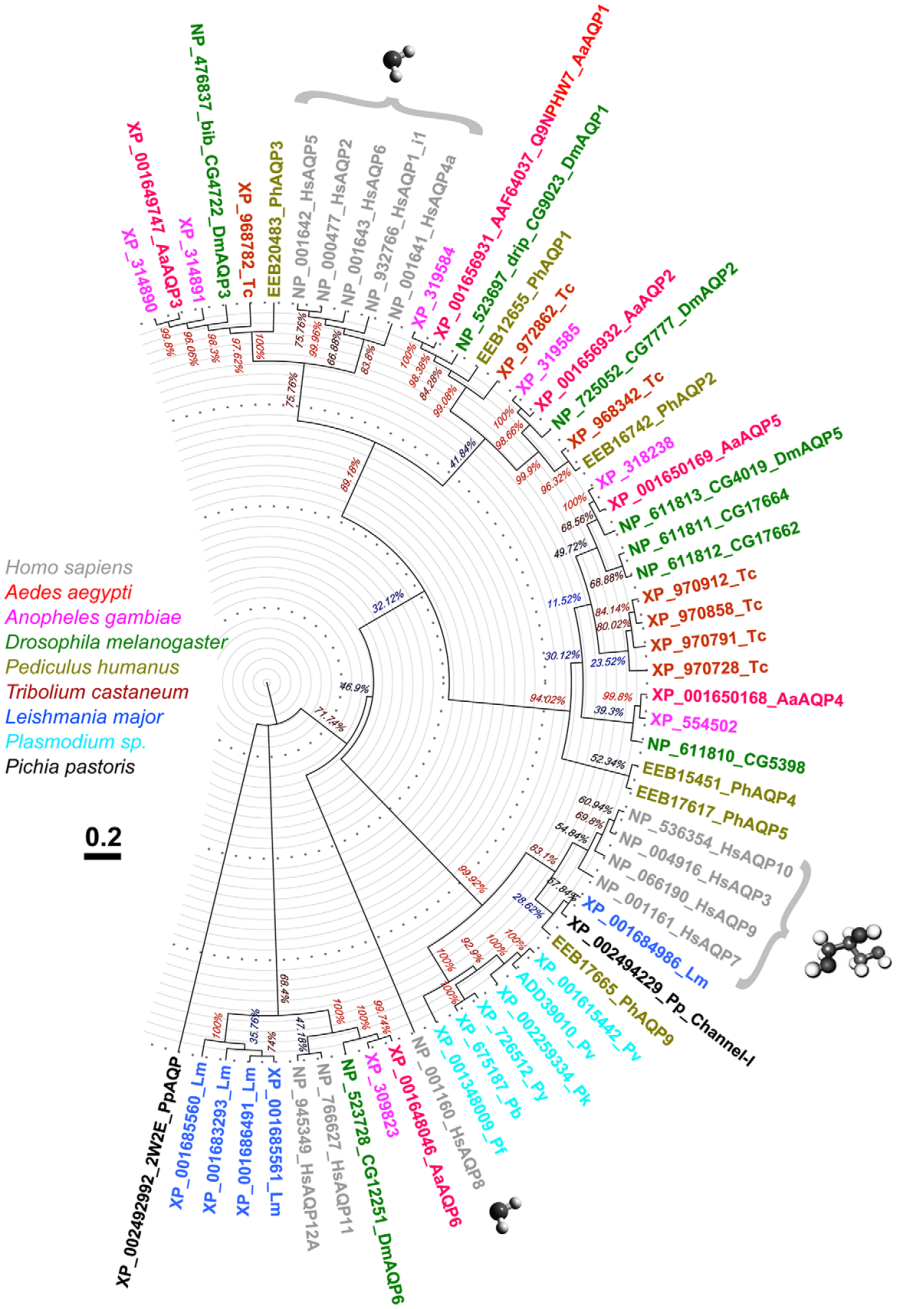
Evolutionary profile of *Ae. aegypti* AQPs. Evolutionary relationships of AQPs from selected organisms with available sequenced genomes. The evolutionary history was inferred using the Neighbor-Joining method [43]. The bootstrap consensus tree inferred from 5000 replicates represents the evolutionary history of the taxa analyzed [44]. The tree is drawn to scale, with branch lengths in the same units as those of the evolutionary distances used to infer the phylogenetic tree. Evolutionary distances were computed using the Poisson correction method [45] and units represent the number of amino acid substitutions per site. The analysis involved 59 amino acid sequences. All ambiguous positions were removed for each sequence pair. There were a total of 236 positions in the final dataset. Evolutionary analyses were conducted in MEGA4 [46]. Initial sequence alignment was completed using PROMALS3D server (PROfile Multiple Alignment with predicted Local Structures and 3D constraints) [37]. The tree is drawn using FigTree software to an approximate 3.7 Bya-long scale with relative branch lengths used to infer the tree. AQPs from different species are color-coded. Confirmed water transporters are labeled with a water molecule, confirmed aquaglyceroporins are labeled with a glycerol molecule.

A phylogenetic tree was constructed to indicate associations among the identified protein sequences (Figure 1). AaAQP1 forms a clade with three proteins from the other insect species and with *Drosophila* DRIP. DRIP represents a typical water transporter [16]. AaAQP2 forms a well defined orthologous clusters with other insect AQPs. AaAQP1, 2, and 3 share a clade with well characterized vertebrate AQP subfamily members that mediate water transport. Within this clade AaAQP3 forms a separate cluster together with *Drosophila* BIB (CG4722), and two *Anopheles* AQPs.

AaAQP4 and AaAQP5 belong to two separate insect-specific clades. Mosquitoes have single representatives in each of these clusters, whereas other insects have apparent gene duplications. There are three relatives of AaAQP5 in fruit fly and four in the flour beetle. AaAQP6 is closely related to *Drosophila* CG12251 and vertebrate AQPs 11 and 12.

### Expression of AQPs in adult female *Ae. aegypti*

**Figure 2:**
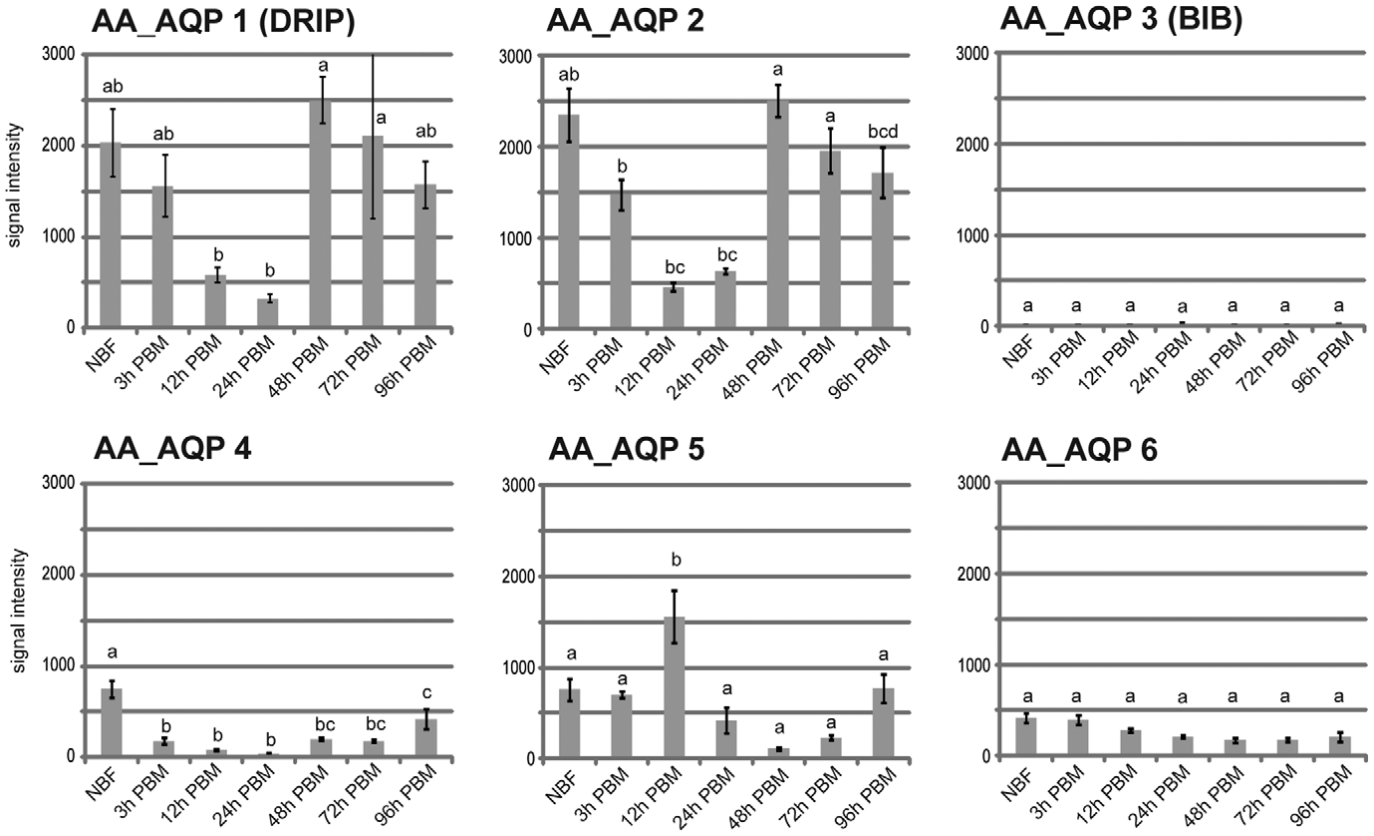
Six *Ae. aegypti* AQPs are expressed in adult female mosquitoes. Overall expression of AaAQPs in adult female mosquitoes during vitellogenesis. Expression was determined by microarray analysis. Six replicates were analyzed, and their mean separated by Tukey–Kramer HSD (p<0.05). Means which share the same letter are not significantly different.

First, we analyzed AQP expression data from microarray assays performed with probes from RNAs isolated from whole mosquito females (Figure 2). As a general trend we found AQP expression down-regulated 12 and 24 h after a blood meal. At the later time points, expression returns to “non blood fed (NBF)” levels. An exception was AaAQP5 which was up regulated 12h PBM but is also down regulated at later time points.

**Figure 3:**
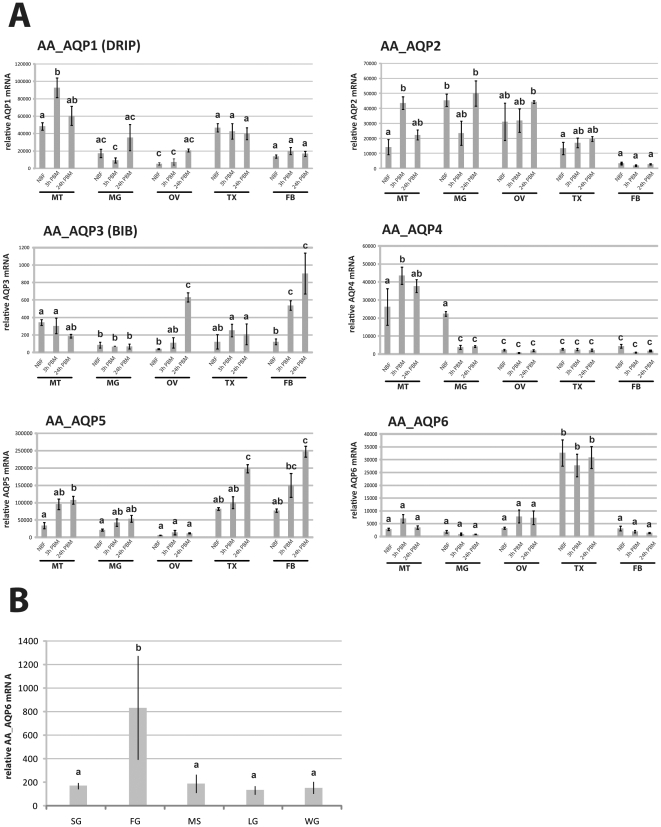
The six *Ae. aegypti* AQP organ/body part expression patterns are highly diverse. **A.** Organ/body part expression patterns. Expression was assayed with q-RT-PCR. The results shown are representative of three separate repeats with similar results. RNA was isolated from organs/body parts of four groups of 20 mosquitoes. MT – Malphigian tubules, MG – posterior midgut, OV – ovaries, TX - thorax, FB – abdominal body wall with fat body. Three replicates were analyzed, and their mean separated by Tukey–Kramer HSD (p<0.05). Means which share the same letter are not significantly different. **B.** Thorax tissue/organ expression pattern of unfed mosquitoes. Relative expression levels were determined via q-RT-PCR. Expression values were normalized with ribosomal protein S7 expression data from the same samples. SG – salivary glands, FG – foregut, MS – wing muscle, LG – legs, WG – wings. Four replicates were analyzed, and their mean separated by Tukey–Kramer HSD (p<0.05). Means which share the same letter are not significantly different.

Next, we determined AQP expression in selected organs and body parts of adult female mosquitoes before a blood meal, 3 and 24 h PBM. Using quantitative RT-PCR, we found that all six *Ae. aegypti* AQPs were expressed in adult female mosquitoes (Figure 3). Different organs/body parts varied considerably in the assortment of AQPs they expressed. The observed patterns of AQP expression were as follows:

#### AaAQP1

This AQP is the mosquito homologue of *Drosophila* DRIP, a highly selective water-specific channel in the fruit fly [16]. AaAQP1 was expressed in all organs and body parts examined with highest expression levels in the MTs. It is significantly up regulated in the MTs 3 h after a blood meal.

#### AaAQP2

We found high AaAQP2 transcript levels it in all organs and body parts except the fat body. It was strongly expressed in the MTs, midgut, and ovaries at all time points. The overall expression pattern during vitellogenesis is similar to AaAQP1. It is significantly up regulated in the MTs 3 h after a blood meal.

#### AaAQP3

This AQP is the homologue of the *Drosophila* BIB. BIB does not function as a water channel in fruit flies but is involved in the regulation of cell adhesion [24]. AaAQP3 was weakly expressed in MTs, midgut, and ovaries and was significantly up regulated in ovaries and fat body PBM.

#### AaAQP4

This uncharacterized AQP was highly expressed in MTs and is significantly up regulated 3 h after a blood meal. It is down regulated in the midgut during early vitellogenesis.

#### AaAQP5

This uncharacterized AQP was highly expressed in all organs except the ovaries and was up regulated in most organs PBM.

#### AaAQP6

This uncharacterized AQP is predominantly expressed in the thorax. The highest mRNA expression levels were found in the foregut (Figure 3B).

### Effect of AQP knockdown on diuresis after PBS injection

**Figure 4:**
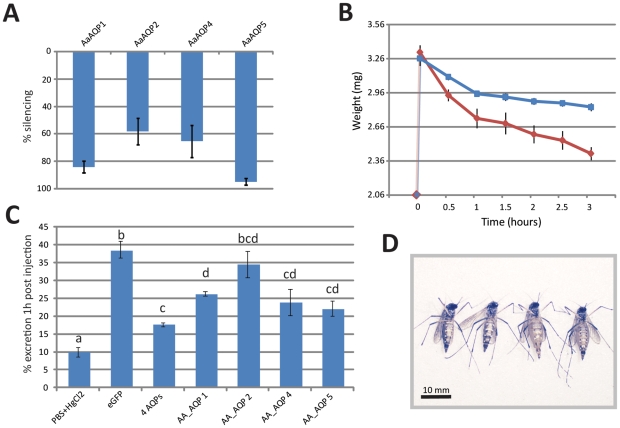
Three AQPs regulate water excretion in *Ae. Aegypti.* **A.** Knockdown efficiency. AQP ds-RNA was injected in mosquitoes and transcript accumulation determined at three days post injection. eGFP dsRNA-injected mosquitoes were used as a control and AQP expression in these mosquitoes was set as 100%. Five groups of ten knockdown mosquitoes were analyzed for each group and the data is expressed as average and standard error **B.** Weight profiles after PBS injection in AQP knockdown and control mosquitoes. Three groups of thirty mosquitoes of a control group (blue) and AQP knockdown group (red, combined knockdown of AQP 1, 2, 4, 5) were injected with 1.25 ul PBS. Average weight was determined before injection (0 point) and in 30 min intervals after injection. **C.** Effect of AQP knockdown on excretion in PBS-injected mosquitoes. Five groups of ten mosquitoes were analyzed for each control and experimental sample. RNAi knockdown was induced three days before the experiment. Replicates were analyzed, and their mean separated by Tukey–Kramer HSD (p<0.05). Bars labeled with the same letter are not significantly different. **D.** Visualization of knockdown effect. *Ae. aegypti* females; 3 h after injection of 2.5 ul PBS. The two mosquitoes on the left are representatives of 20 control mosquitoes that received eGFP dsRNA injections. The two mosquitoes on the right are representatives with combined knockdown of AQP1, 2, 4, and 5.

RNAi-mediated gene expression knockdown was determined in samples of total RNA isolated from MTs dissected three days after injection of AQP dsRNA. Knockdown was successful in the mosquito MTs with efficiencies between 95% (AaAQP5) and 60% (AaAQP2) reduction in transcript accumulation (Figure 4A).

In order to determine how fast PBS-injected mosquitoes start discharging urine, we injected female mosquitoes with 1.25 ul PBS and observed them under a stereomicroscope to determine the time period till the onset of urine discharge. Injected females started to discharge urine droplets on average at 157 s (SE  = ±16 s) after injection, thus leaving a window of two minutes to perform the first weight measurement after injection. Excretion was strongest in the first hour after injection (Figure 4B, control group, blue line).

To test the hypothesis that the AQP proteins are contributors to the function of the MT, we generated knockdown mosquitoes for each MT-expressed AQP transcript by dsRNA injection and tested for urine production using our in vivo diuresis assay (Figure 4C). Negative control mosquitoes (injected with a dsRNA directed against the unrelated jellyfish protein eGFP) excreted 39% of the injected 1.25 ul PBS within one hour. In contrast, positive control mosquitoes injected with 1.25 ul of 200 uM HgCl_2_ in PBS (known to suppress AQP activity [13]) excreted only 10% in the first hour after injection.

AaAQP1, 4, and 5 knockdown mosquitoes showed significantly reduced excretion rates compared to the eGFP dsRNA injected control (Figure 4C). Knockdown of AaAQP2 did not result in a significant effect. The strongest effect of a single gene knockdown was observed after knocking down AaAQP5 which resulted in a decline of excretion to only 22%. The combined knockdown of all four MT-expressed AQPs reduced excretion to only 18% of the injected PBS.

In order to visualize the effect of the combined knockdown on diuresis we injected control and combined knockdown mosquitoes with 2.5 ul PBS. Figure 4D shows two representatives of each group 3 h after injection.

## Discussion

This report is the first comprehensive study on AQP genes and their expression in the adult yellow fever mosquito, *Aedes aegypti*. We specifically focused on AQPs expressed in the MTs, an insect organ, specialized in water and waste excretion.

An interesting result of our phylogenetic analysis (see Figure 1) is that we were unable to identify any typical aquaglyceroporin in dipteran insects. Human aquaglyceroporins, the *Homo sapiens* AQPs 3, 7, 9, and 10, form a separate clade with six *Plasmodium*, one *Leishmania*, one fungal and one louse AQP. A multiple sequence alignment of all AQPs used for our analysis (Figure S1) revealed that all aquaglyceroporins share a cysteine residue at position six, C-terminal to the NPA motif in the B-loop. None of the dipteran AQPs in our analysis had a cysteine at this position. The fact that dipteran insects do not appear to possess an AQP-like glycerol transporter raises the question as to how they transport glycerol over cell membranes. Glycerol plays an important role in insect cold tolerance and diapause and is found in high concentration in the hemolymph of diapausing insects [25].

The microarray expression data was created with RNA isolated from total mosquitoes (Figure 2) and shows that AQP expression was generally down regulated after a blood meal, even at 3 h PBM. After taking a blood meal mosquitoes seek out a resting place to digest the blood and perform vitellogenesis and egg development. This resting phase extends over a period of approximately two days. During this time mosquitoes don't take up water, therefore high AQP expression levels are not necessary.

AQP expression show specific patterns in distinct organs/body parts (Figure 3). All examined organs/body parts differ in their AQP expression patterns. We were specifically interested in patterns from midgut and MTs since blood meal-derived water has to cross the midgut epithelium and subsequently the MT epithelium for excretion. AQPs 1, 2, 4, and 5 were expressed in the midgut. Interestingly, three AQPs expressed in the midgut were down regulated at the 3 h PBM time point, corresponding to the time at which the bulk of blood meal-derived water has already been excreted. Our data suggests that AQPs 1, 4, and 5 are the principal AQPs in the MTs of adult females.

We developed an *in vivo* diuresis assay for adult yellow fever mosquito females based on PBS injection. PBS is a non-toxic isotonic buffer containing sodium chloride, sodium phosphate, potassium chloride and potassium phosphate. This novel assay has several advantages over classical blood meal-based assays. Firstly, mosquitoes receive a standardized amount of PBS, while blood meal sizes can vary significantly. Secondly, since *Ae. aegypti* mosquitoes start secreting urine about 30 s after starting a blood meal [1], [26], the determination of blood meal sizes is difficult. Applying our method, mosquitoes injected with PBS started secreting urine after 2 min, which allowed accurate measurement of their weight after injection. As mentioned above, mosquito diuresis is controlled by neuropeptide hormones that are secreted from the central nervous system in response to unknown stimuli associated with blood ingestion. PBS injection triggers this response efficiently. The rapid onset of diuresis after injection suggests that AQP activity in the MT is regulated by trafficking of AQP-containing vesicles to the plasma membrane, analogous to the processes described for the renal collection duct in human kidney [27].

RNAi is a powerful tool used to study gene function in mosquitoes. We successfully employed RNAi-mediated knockdown for the analysis of AQP function in the MT. Knockdown rates, as determined by real time PCR, were between 60 and 95% and therefore well inside the range that can be achieved by dsRNA transfection in cell culture [28]. Similar knockdown efficiencies have been found in whole mosquitoes [29].

A point of concern is that the AQP dsRNA injections likely resulted in AQP knockdown in tissues other than the MTs. However, excreted watery liquids have been shown to always pass through the MT in adult mosquitoes [1], [5], [30]. Therefore we expect the effects of AQP knockdown in other tissues to be negligible for the outcomes of our *in vivo* diuresis experiments.

As a positive control for successful AQP inhibition we used mercury ions (Hg^2+^), which are capable of binding with a cysteine and an alanine within the AQP pore, thus obstructing water transport through the channel. Mosquitoes injected with 200 uM HgCl_2_ were still able to excrete 10% of the injected fluid in one hour. This might be due to paracellular permeability, water transport through the space between the cells, which has been described in insect MTs and can be enhanced by kinins [26].

Using RNAi knockdown mosquitoes we confirmed the function of three different AQPs in the MTs of *Ae. aegypti*. While RNAi control mosquitoes were able to excrete about 40% of the injected fluid in one hour, knockdown of single AQPs (AaAQP1, 4, or 5) resulted in a significant decrease in excretion. Simultanous knockdown of all four MT-expressed AQPs reduced excretion down to 18% of the injected fluid in one hour, indicating that a combination of AaAQPs 1, 4, and 5 performs water transport in the MTs of female *Ae. aegypti*.

The redundancy of function we have observed here has also been noted in human kidney where seven different AQP proteins are expressed [31]. One plausible explanation would be that several AQP genes with different promoters allow the fine regulation of AQP expression in stage- and tissue specific manner or after a range of stimuli.

There is a great need for the development of novel, effective insecticides to fight insect vectors, since the public health insecticides currently in use are based on only a limited number of active compounds [32]. Because of their vital importance in insect larvae and adult homeostasis, insect AQPs could become targets for the development of novel insecticides. The study presented here has identified six genes encoding putative AQP membrane transporters in *Ae. aegypti* and demonstrated the functional role of three of them in regulation of water transport. Further analysis of these AQPs and their regulation has the potential to contribute to the future development of novel anti-vector strategies.

## Materials and Methods

### Mosquito rearing

The *Ae. aegypti* mosquito strain UGAL was maintained in laboratory culture as has been previously described by Hays and Raikhel [33]. The strain was reared at a temperature of 28°C with 80% humidity and a photoperiod of 14 h light and 10 h dark. Larvae were fed on a diet of ground rat food, yeast and albumin (1∶1∶1 w/w).

### Sequence Identification & Phylogenetic analysis

Predicted cDNA and deduced AA sequences of AQP family members were identified using BLAST at three databases: Ensembl [34], Genbank [35], and VectorBase [36]. A sequence alignment (supplemental online material: Figure 1) was performed using PROMALS3D software which considers structural constraints for the divergent AQPs protein sequences [37]. The reconstruction of the phylogenetic tree was performed using Mega 4 [38]. The tree was visualized using FigTree software [39].

### Microarray Expression Studies

Three biological samples composed of four day old mosquitoes were collected consisting of a pool of 20 insects. Four day old females were blood fed and three biological samples (20 females each) were collected at several times after the blood meal. Total RNA was extracted with TRIzol® (Invitrogen, Carlsbad, CA) and further purified with Qiagen RNAeasy columns (Qiagen,Valencia, CA) with DNase treatment according to the manufacturer's recommendations. RNA quality was assessed by capillary electrophoresis using the Agilent Bioanalyzer 2100 and spectrophotometric analysis. The RNA was reverse transcribed and amplified using WT Ovation Pico (Cat# 3300-60, NuGen, San Carlos, CA) and 2 g of this cDNA was labeled with Cy3 (Cat# 5190-1305, Agilent technologies, Santa Clara, CA) according to the Agilent Oligonucleotide Array-Based CGH For Genomic DNA Analysis Protocol (Cat# G4410-90010, Agilent technologies, Santa Clara, CA). Hybridization, washing, and scanning were processed as per the Agilent OneColor Microarray-Based Gene Expression Analysis Protocol (Cat# G4140-90040, Agilent technologies, Santa Clara, CA). Expression signals were normalized for background within chips with the Agilent spatial correction algorithm and were normalized between chips using quantile normalization. The data is available at Gene Expression Omnibus (GEO, www.ncbi.nlm.nih.gov/geo/) under series record GSE22339 [40]. Statistical analysis was performed with InStat (GraphPad Software, La Jolla, CA).

### qRT-PCR Expression Studies

Gene-specific primers were developed using Primer BLAST [41]. Total RNA was obtained from different larval stages, pupae, and adult females with TRIzol® solution. Tissue-specific RNAs were isolated after dissection of samples from 30 individual mosquitoes including previtellogenic females 72 h after eclosion and female mosquitoes 3 h and 24 h post blood meal. Transcript was analyzed and quantified using quantitative RT-PCR (qPCR) using iQ Supermix (Biorad, Hercules, CA). Primers were as follows: AaAQP1f: ACC GGC ATC AGA AAG GAG AAG CG; AaAQP1 r: GCC TGC TGT TTG ATG TGT TGT GCA; AaAQP2 f: GCT CGC TCG TTT GGA ACG GC; AaAQP2 r: CAC GGT AGC GCT CTG AGG CG; AaAQP3 f: AGG TCC AGT GGG GAT GGC CC; AaAQP3 r: CAG CTG AGG TGG TGG CGG TG; AaAQP4 f: CTG CCG CCT GCA GTG TGG AA; AaAQP4 r: TGT GGA AGT TCT CGT CGG AAG ACG T; AaAQP5 f: CGG TGT TCA GGC GCG AGG TT; AaAQP5 r: ATC CCG CGT TGG TGG AAC GG; AaAQP6 f: CAG CTT GGT CGT GGC CGC AT; AaAQP6 r: GCA CAG CTC CCG CAC ACG AT.

### RNAi knockdown experiments

Generation of double-stranded RNAs (dsRNA) was performed as described earlier [42] using primers with the T7 primer sequences attached. PCR product was used as template for dsRNA synthesis with the MEGAscripts T7 Kit (Ambion, Austin, Tx). Approximately 500 ng of dsRNA in 268 nl of pyrogen-free H_2_O was injected into the thorax of CO_2_-anesthetized female mosquitoes three days after adult emergence. The injected mosquitoes were then allowed to recover for 3 days before diuresis assay. Knockdown effectiveness was confirmed with qPCR. Primers were as follows (The T7 sequence is omitted): AaAQP1f: TAA TAC GAC TCA CTA TAG GGA GCA CTA TGG GCT GGG GCG GAG ACT; AaAQP1 r: TAA TAC GAC TCA CTA TAG GGA CGG CTG GTC CGA AAG AGC GAG CTG; AaAQP2 f:TAA TAC GAC TCA CTA TAG GGA CTG CTG GCT TAC TTG CGG CTG GCA; AaAQP2 r: TAA TAC GAC TCA CTA TAG GGG CTA CAC GGT AGC GCT CTG AGG CGG; AaAQP3 f: TAA TAC GAC TCA CTA TAG GGC CCC ATC CCC AAG CGG GTG AAC CAC; AaAQP3 r: TAA TAC GAC TCA CTA TAG GGT GGG CCA TTG GGTAGC CCC CTG GAT; AaAQP4 f: TAA TAC GAC TCA CTA TAG GGG CGG CAT CGG GTT CGG CTT CAC AGT; AaAQP4 r: TAA TAC GAC TCA CTA TAG GGT GGC CGG GTT CAT ACT CGC TCC GGT; AaAQP5 f: TAA TAC GAC TCA CTA TAG GGT ACG TTG CGG CCC AGT GCA TCG GAG; AaAQP5 r: TAA TAC GAC TCA CTA TAG GGG GAA CCT CGC GCC TGA ACA CCG TCT; AaAQP6 f: TAA TAC GAC TCA CTA TAG GGG ATC GCG GCA GTT GCT CGC CGA GTG; AaAQP6 r: TAA TAC GAC TCA CTA TAG GGA GGC CAA GCG ACA GCA CAG AGT GGC.

### In vivo diuresis assay

Knockdown and control (eGFP dsRNA-injected) mosquitoes were anesthetized with CO_2_ and pooled into groups of ten. Group weight was determined on a precision weighing balance. Each mosquito was subsequently injected with 1.25 ul of PBS and the groups were immediately weighed. Diuresis was monitored by weighing mosquitoes in 30 min intervals. Control mosquitoes were injected with 1.25 ul 200 uM HgCl_2_ in PBS. After the last weighing the MTs were dissected and RNA was isolated in order to determine the knockdown efficiency.

## Supporting Information

Figure S1 **Comprehensive alignment of 72 aquaporin family members.** Sequences are from *Ae. aegypti*, *Anopheles gambiae*, *Drosophila melanogaster*, *Pediculus humanus*, *Tribolium castaneum*, *Homo sapiens*, *Leishmania major*, *Plasmodium sp.*, *and Pichia pastoris.* Darker patterns represent higher sequence similarity.
(PDF)

Table S1 ***Ae. aegypti*** **aquaporins.** ^1^ AAEL – VectorBase; all others – NCBI Genbank; ^2^ [17]; ^3^ The BIB cluster is duplicated in the *Anopheles* genome; ^4^ This cluster is expanded in the fruit fly genome.
(DOC)
