# Supplementary figures and images for: The Aquaporin Gene Family of the Yellow Fever Mosquito, Aedes aegypti
Source: PLoS One. 2010 Dec 29;5(12):e15578. doi: 10.1371/journal.pone.0015578 (PMC3014591; doi:10.1371/journal.pone.0015578)

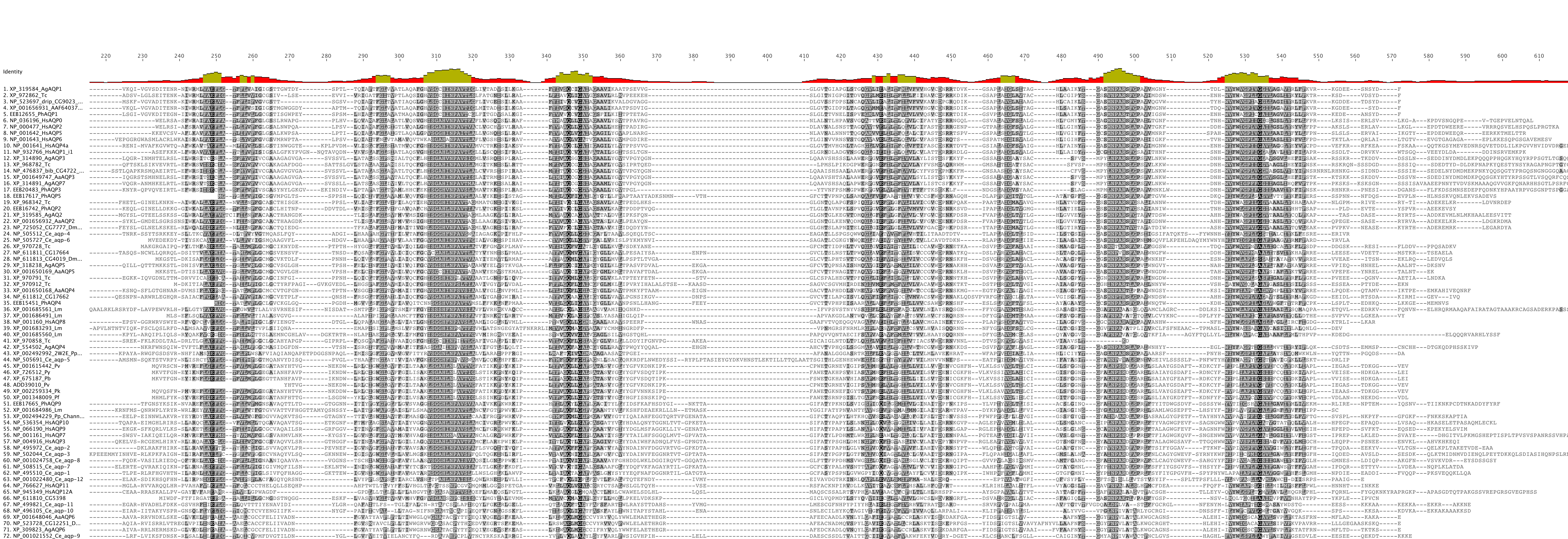

Supplement: Figure S1 — Comprehensive alignment of 72 aquaporin family members. Sequences are from Ae. aegypti, Anopheles gambiae, Drosophila melanogaster, Pediculus humanus, Tribolium castaneum, Homo sapiens, Leishmania major, Plasmodium sp., and Pichia pastoris. Darker patterns represent higher sequence similarity. (PDF) [file pone.0015578.s001.pdf]
