# Supplementary material for: The Aquaporin Gene Family of the Yellow Fever Mosquito, Aedes aegypti
Source: PLoS One. 2010 Dec 29;5(12):e15578. doi: 10.1371/journal.pone.0015578 (PMC3014591; doi:10.1371/journal.pone.0015578)
Supplement: Table S1 — Ae. aegypti aquaporins. 1 AAEL – VectorBase; all others – NCBI Genbank; 2 [17]; 3 The BIB cluster is duplicated in the Anopheles genome; 4 This cluster is expanded in the fruit fly genome. (DOC) [file pone.0015578.s002.doc]

| **Name** | **Accession numbers^1^** | **Length** | **putative function** | ***Ag* Homologs** | ***Dm* homologs** |
| --- | --- | --- | --- | --- | --- |
| AaAQP1  DRIP^2^ | AAEL003512  XP_001656931  AAF64037  Q9NHW7 | 244  249 | Aquaporin | XP_319584 | CG9023 (drip) |
| AaAQP2 | AAEL003550  XP_001656932 | 264 | Aquaporin | XP_319585 | CG7777 |
| AaAQP3  BIB^3^ | AAEL004741  XP_001649747 | 616 | Ion channel/ cell adhesion factor^4^ | XP_314890 | CG4722 (bib) |
| AaAQP4 | AAEL005001  XP_001650168 | 292 | Aquaporin | XP_554502 | CG5398 |
| AaAQP5^4^ | AAEL005008  XP_001650169 | 249 | Aquaporin | XP_318238 | CG4019 |
| AaAQP6 | AAEL014108 AAEL014255  XP_001648319  XP_001648046 | 261 | unknown | XP_309823 | CG12251 |
